# Spatio-temporally modulated fluorescence imager for intraoperative nerve identification

**DOI:** 10.1117/1.JBO.31.10.106001

**Published:** 2026-10-01

**Authors:** Jesung Park, Hyejin Yoo, Samuel S. Streeter, John Grimble, Mark T. Scimone, Gopi Maguluri, Jeffrey L. Campbell, Madelyn O’Shaughnessy, Nicusor Iftimia, Eric R. Henderson, Brian W. Pogue, Youbo Zhao

**Affiliations:** a Physical Sciences, Inc., Andover, Massachusetts, United States; b Dartmouth College, Thayer School of Engineering, Hanover, New Hampshire, United States; c Dartmouth College, Geisel School of Medicine, Hanover, New Hampshire, United States; d University of New Hampshire, Life Sciences, Biotechnology, Manchester, New Hampshire, United States

**Keywords:** fluorescence-guided surgery, nerve-labeling agents, intraoperative nerve identification, spatiotemporal modulated imaging

## Abstract

**Significance:**

Fluorescence-guided surgery (FGS) based on nerve-labeling agents has the potential to enhance nerve identification during various operational procedures including orthopedic, craniofacial, and otolaryngological surgeries that involve complex and delicate nerve structures. A major challenge in FGS is the adverse impact of strong ambient light, which interferes with weak fluorescence signals, especially at visible wavelengths. Currently, this issue is mitigated by dimming or turning off the operating room (OR) lights during the fluorescence imaging procedure. However, this causes substantial disruption to the surgical workflow and reduces the potential for clinical adoption of FGS technologies.

**Aim:**

In this work, we develop a novel intraoperative fluorescence imager that is based on a spatio-temporal modulation (STM) approach to suppress interference from strong OR lights.

**Approach:**

The STM technology employed a line-scan imaging scheme that encodes the fluorescence signal into a distinctive spatial frequency within a narrow time window. By applying spatial frequency demodulation and time-gating methods, the imager effectively extracted the fluorescence signals by rejecting temporally constant and spatially uniform background OR lights.

**Results:**

The performance of the imager in detecting nerve structures in realistic OR lighting conditions was validated during *in vivo* animal studies. Additional evaluation using *ex vivo* human nerve tissue specimens and perfused amputated human limbs further supported the clinical suitability and readiness.

**Conclusions:**

This innovative imaging technology enables high-contrast fluorescence visualization of nerve tissue under standard OR lighting and represents a significant step toward practical clinical implementation of FGS for enhanced nerve identification.

## 1 Introduction

Unintended nerve injury during surgery may lead to significant morbidity and mortality.^1^^–^^4^ This frequently overlooked surgical complication affects millions of patients worldwide, causing chronic pain, numbness, loss of function, and peripheral neuropathy. Avoiding nerve injury is of particular significance in many orthopedic and otolaryngological surgeries dealing with nerve-rich soft tissues, especially the limbs and craniofacial structures where dense networks of sensory and motor nerves are present. Orthopedic surgery nerve damage is a reported complication in 15% of the total medical liability claims in the United States.^5^^–^^7^ Current nerve identification methods based on gross visual appearance and assumptions about anatomical location are subject to error and may lead to injury, especially for nerves that are thin, buried, or aberrant.^8^ Technologies recently evaluated to assist in intraoperative nerve identification include electromyography, ultrasound, optical coherence tomography, and confocal endomicroscopy.^9^^–^^11^ However, these techniques lack specificity, resolution, or wide-field sensing, all required for precise and real-time nerve detection during surgery. Thus, distinctive visualization of nerve tissue from adjacent structures remains challenging and a major contributor to surgical nerve injuries.

Fluorescence imaging has emerged as a highly promising approach for image-guided surgical applications.^12^^–^^16^ Several fluorescence-image-guided surgical (FGS) systems, including FLARE™ by Curadel, Fluorobeam by Fluoptics, daVinci robotic systems by Intuitive Surgical, SPY systems by Novadaq, Photodynamic Eye by Hamamatsu, and Artemis by Quest Medical, have become commercially available for intraoperative imaging of fluorescence-labeled tissues.^17^^,^^18^ These systems primarily operate in the near-infrared (NIR) spectrum, which is optimal for deep tissue imaging and minimizes interference from ambient light typically present in the operating room (OR).^17^^–^^19^ However, this focus on NIR wavelengths limits their suitability for nerve imaging applications that utilize visible spectrum fluorophores. Under standard surgical lighting conditions, these tissue-specific fluorescence signals are susceptible to being overwhelmed by intense ambient light, making it difficult to achieve high-contrast visualization of nerve tissue. As a result, the available clinical FGS systems, designed primarily for NIR imaging, cannot be readily adapted for nerve-specific FGS.

Recently developed nerve-labeling fluorescent agents, such as fluorescein-labeled bevonescein,^20^ Hsp1a with BODIPY-FL,^21^ FAM–NP41,^13^^,^^22^ distyrylbenzene (DSB)^14^^,^^15^^,^^23^ and Oxazine derivative fluorophores^17^ are primarily in the visible or NIR.^24^ For most open-cavity fluorescence-guided surgery (FGS) applications, effectively eliminating the negative interference caused by strong ambient light in the OR is critical,^16^^,^^25^^–^^30^ yet highly challenging. The low intensity of tissue-specific fluorescence signals makes them particularly vulnerable to interference from the background, which can greatly diminish image contrast and, consequently, the utility of the FGS data. A recent study highlighted this limitation by demonstrating that strong surgical lighting, whether LED or halogen, can reduce imaging performance by approximately three-fold in comparison to dark conditions.^31^ This issue is especially problematic in scenarios involving low-dose administration, quantitative fluorescence analysis, or the identification of subsurface and fine tissue structures, where the signals are inherently weak. Additionally, it is a concern in multi-wavelength fluorescence imaging, as some fluorophores may need to operate at shorter wavelengths, further exacerbating the interference from ambient light.^24^^,^^27^^–^^29^

The standard method currently employed to lessen the impact of ambient room lighting is to dim or switch off the lights during FGS imaging.^25^^–^^27^^,^^32^^,^^33^ While this can help enhance the visibility of fluorescence signals, it introduces considerable disruption to the normal surgical workflow. Such disruptions can decrease the enthusiasm of surgeons, operating room staff, and hospital administration for adopting the technology and ultimately hinder its broader clinical implementation. Other approaches to suppress ambient light interference include spectral filtering^34^^,^^35^ and time-gating techniques.^27^^,^^30^^,^^33^^,^^36^ Using narrow spectral filters to block out-of-band background light is particularly efficient for near-infrared (NIR) imaging, as most ambient light in the OR is concentrated in the visible spectrum. However, these filters may also block some portions of the fluorescence emission, thereby reducing the overall signal utilization efficiency. Time-gated imaging is another common strategy, with several related patents issued in this area.^33^^,^^37^^–^^39^ Nevertheless, the effectiveness of background rejection in time-gated imaging is significantly constrained by the duty cycle of the pulsed light source. Without the use of pulsed laser systems, it is difficult for time-gated imaging to eliminate more than 99% of the ambient light. Given the high intensity of white-light illumination in the OR, which reaches $>1\;\mathrm{mW}/{\mathrm{cm}}^{2}$ at visible wavelengths compared to useful fluorescence signal levels as low as $1\;\mathrm{nW}/{\mathrm{cm}}^{2}$, even minimal amounts of ambient light can severely compromise imaging results. Therefore, reliably detecting fluorescence signatures from imaging agents in the visible spectrum under standard OR lighting conditions remains a substantial challenge for current FGS technologies.

Here, we report the development of an FGS system that can efficiently acquire weak nerve-specific fluorescence signals at visible wavelengths in the presence of strong ambient light. To overcome the limitations associated with background interference of FGS imaging, a line-scan-based spatio-temporal modulation (STM) fluorescence imager was developed and evaluated using fluorescently labeled murine sciatic nerves *in vivo* and human nerve tissues *ex vivo*. This STM technology provides a viable solution for reliable acquisition of weak fluorescence images in the presence of strong ambient light (e.g., $10^{5}\times$ brighter than the fluorescence signal), eliminating the inconvenient requirements of dimming OR light during FGS.

## 2 Materials and Methods

### 2.1 Principle of Line-Scan-Based STM

Time-gated detection is a commonly used method to detect weak signals under strong continuous background. As shown in Fig. 1(a), the signal-to-background ratio (SBR) of a time-gated approach is inversely proportional to the duty cycle (Δt/T) of the light source. To achieve a high SBR, a low duty cycle source is required. However, for typical light sources, such as light emitting diodes (LEDs) with limited peak power, a low duty cycle leads to low average power and thus to a low signal level. As a result, time-gated imaging using pulsed LED sources cannot achieve background suppression better than 100:1. This issue may be solved by using pulsed lasers, such as Q-switch or mode-locked lasers; however, the use of such high intensity lasers in clinics raises significant safety concerns.

**Fig. 1:**
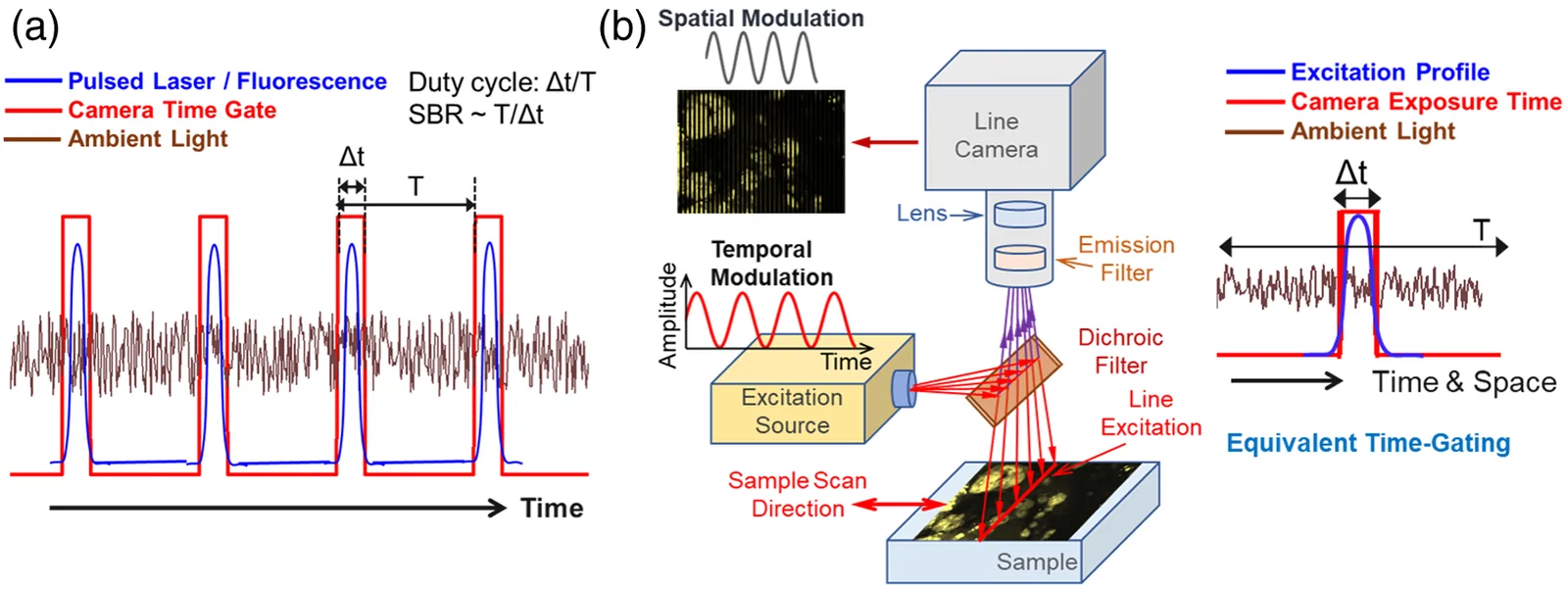
Principle of the time-gated detection scheme to improve signal to background ratio (SBR) (a), and spatial modulation and equivalent time-gating effect of the line-scan imaging approach (b).

Our approach to mitigate this issue is to use a line-scan imaging approach combined with modulated illumination. The line-scan approach is essentially a time-gated detection while the temporal modulation converts into spatial modulation in the line-scan imaging scheme. As shown in Fig. 1(b), during line-scanning, each exposure of the line camera is essentially time-gated. This is because each line scanned across the sample is recorded sequentially, meaning each line of pixels is acquired at a distinct, well-defined moment in time. The system selectively detects signals from specific spatial regions within defined temporal windows, effectively gating or restricting signal detection in time. As a result, for each line, the signal integration time is N times shorter than when a 2-D camera is used, which stares at the entire field during the camera exposure time [e.g., T in Fig. 1(b)], where N is the number of cycles across each illumination line. Therefore, the sequentially and temporally defined acquisition inherent to line-scanning techniques effectively renders them a form of time-gated imaging, in which excitation at each line position is gated with a duty cycle of 1/N. In this case, the gating efficiency (Δt/T) is equivalent to 1/N. Achieving similar gating efficiency and thus background rejection ratio (e.g., >99.9%) would otherwise be difficult by reducing the duty cycle of the laser source. We note that this time-gating effect does not require pulsing the excitation light at a low duty cycle.

The spatial modulation is formed as the excitation laser is modulated in time while the line focus is scanned across the field [Fig. 1(b)]. As shown in Fig. 2, the spatial modulation (altering black/white stripes) shifts the spatial frequency components of the fluorescence image away from those of the white (ambient) light image which is spatially uniform. Therefore, in the Fourier domain, the modulated fluorescence and white-light background can be separated. Due to the symmetric property of the Fourier pattern, complete information of the fluorescence image is contained in the semicircles, and thus the fluorescence image can be reconstructed by an inverse Fourier transformation.

**Fig. 2:**
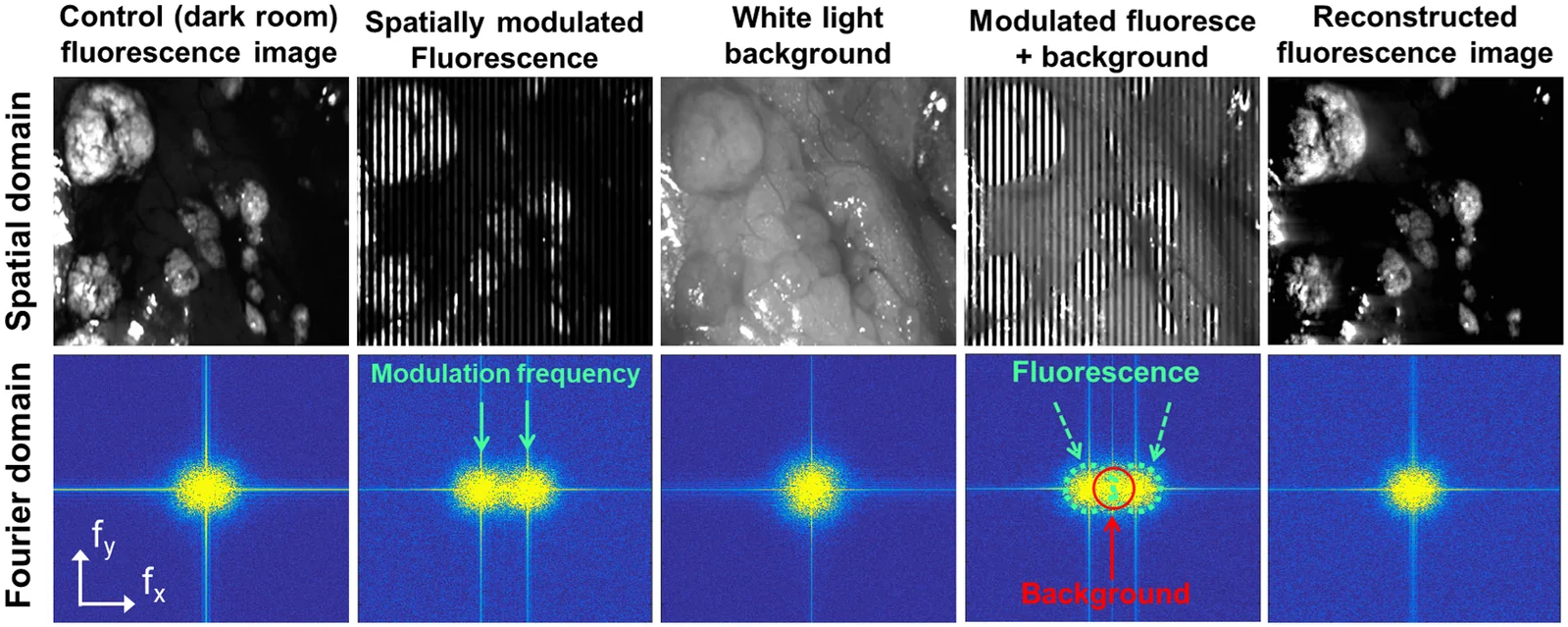
Principle of spatially modulated (structured) illumination to separate fluorescence from ambient background in Fourier domain. Example images of ovarian tissue (upper panel) and corresponding Fourier spectra (lower panel). All the images used in this data were extracted from Ref. 12, reproduced with permission from Springer Nature.

Although spatially modulated illumination has been used in other imaging modalities such as Fourier domain imaging (FDI)^40^^–^^42^ and super-resolution structured illumination microscopy (SIM),^43^^,^^44^ we note that this STM approach uniquely applied the structured illumination to eliminate the ambient light background. In FDI of biological tissues, several spatially modulated illumination patterns are used to detect the modulation depths at different spatial frequencies, which are related to intrinsic optical properties of the tissue (e.g., optical absorption and optical scattering). In the STM imager, instead, only one modulation pattern is used to shift the spatial frequency contents of the fluorescence image in the Fourier domain, and the modulation depth information is discarded. During SIM imaging, multiple (at least three) exposures of differently oriented illumination patterns are used to expand the frequency bandwidth and thus to improve the spatial resolution of the imaging system. However, the goal of the current STM approach is not to achieve super-resolution, but rather to use a single exposure to separate fluorescence image information from the ambient light background.

To achieve efficient background rejection, a high modulation depth of the structured illumination is critical. However, the modulation depth can be degraded when the modulation frequency is close to the spatial resolution limit due to the decay of the optical transfer function of the optical system on the edge of the spatial frequency band.^45^^,^^46^ Light scattering by tissue structures can also reduce the modulation depth. These effects lead to a tradeoff between spatial resolution and background rejection rate. Here we leverage the line-scan imaging approach to mitigate this issue. As shown in Fig. 1(b), the line-scan approach converts a sinusoidal temporal modulation into a spatial modulation to the final 2-D image. In this case, the modulation depth (or contrast) is arbitrarily high, limited only by the amplitude modulation of the excitation source, independent of the spatial frequency bandwidth of the system and the sample properties (e.g., tissue optical absorption and/or scattering). This benefit enables high-efficiency rejection of ambient light background.

The STM approach is conceptually related to spatial frequency domain imaging (SFDI), as both techniques employ spatially modulated excitation and demodulation to separate the encoded imaging signal from the background. Recent SFDI implementations have further incorporated temporal modulation or time-gated acquisition to suppress ambient light interference, either by extracting temporally encoded signals using per-pixel Fourier demodulation^47^ or by rapidly acquiring a dedicated ambient-light image for direct background subtraction.^48^ These approaches generally require synchronized modulation of the illumination and detector, and some implementations rely on specialized high-speed or multi-tap image sensors. In contrast, the STM approach converts the temporal modulation of a scanned excitation line into spatial modulation in the reconstructed 2-D image. This preserves high modulation contrast because it is determined mainly by the laser’s modulation efficiency and is less affected by the optical transfer limitations of the imaging system or tissue-induced fringe blurring. Unlike SFDI, where modulation loss provides information about tissue optical properties, STM uses the preserved modulation for efficient Fourier-domain separation of fluorescence from ambient background. This implementation can be achieved using a conventional scientific CMOS camera operated in line-readout mode, without requiring a custom multi-tap CMOS sensor. In addition to frequency-domain separation of the fluorescence signal, the short line-detection window reduces the integration of ambient light at each spatial location. Furthermore, the line-scanning geometry provides a quasi-confocal effect that suppresses out-of-focus and multiply scattered fluorescence, thereby improving image contrast under strong ambient lighting conditions.

### 2.2 Instrument Description

The optical design and schematic of the STM fluorescence imager are shown in Figs. 3(a) and 3(b), respectively. By leveraging the ability to separate imaging signals in the Fourier domain, we were able to image two wavelength channels to distinguish both nerves and blood vessels from the tissue. To achieve this, two fiber-coupled laser diodes, one at 637 nm [LP637-SF70, Thorlabs, Newton, New Jersey (NJ), United States] and the other at 785 nm (LP785-SF100, Thorlabs, Newton, NJ) were used. These two lasers were selected to excite a nerve-specific fluorescent agent (i.e., LGW16-03, an Oxazine4 derivative,^49^ with peak excitation/emission of 626/655  nm in phosphate buffered saline (PBS)), and a NIR fluorescence agent in the common 800 nm fluorescence channel, such as indocyanine green (ICG), which is frequently used clinically for mapping blood vessels (peak excitation/emission of 800/825  nm in plasma^50^). Two laser beams were combined using a fiber-based 640/785  nm wavelength-division multiplexer (WDM) (NR74F1, Thorlabs, Newton, NJ). The output of the excitation laser beam was first collimated and then focused by a cylindrical lens (f=10  mm, LJ1878L1-A, Thorlabs, Newton, NJ) to form a line-shaped beam in the back focal plane of the objective in the direction perpendicular to the scan direction. A custom-designed micro-prism (0.9 mm width) was used as a strip mirror to separate the line-shape excitation beam and the circular fluorescence signal that goes around the narrow strip mirror, eliminating the need for a dichroic mirror for the separation of the excitation and the fluorescence light beams.^51^ This line-shaped beam was scanned across the sample using a galvanometer (QS10Y-AG, Thorlabs, Newton, NJ). The back-propagated (epi-detection) fluorescence signal from the sample was collected using the same objective, and then separated by a short-pass filter (FELH0650, Thorlabs, Newton, NJ). The short-pass filter functioned as a dichroic mirror with a cutoff wavelength shifted to ∼625  nm at an incident angle of ∼45  deg to separate the visible and fluorescence channels. In the fluorescence channel, the back-propagated fluorescence signal was focused by an achromatic lens (f=60  mm, AC254-060-A, Thorlabs, Newton, NJ) and collected using a high-sensitivity scientific CMOS camera (CB042 MG-GP-BSI, Ximea, Lakewood, CO). This was a compact, scientific grade camera that has a back-illumination sCMOS chip to provide high quantum efficiency (>90% at the peak wavelength) and low readout noise ($1.6\times 10^{-1}/\text{pixel}/\text{frame}$), and thus a high signal to noise ratio (SNR). Although this is an area camera, it can be operated in line-scan mode by reading only a few lines at high speeds, e.g., 150 kHz at 1 line. We have chosen to operate at 20 kHz for 4 lines for optimal performance. A turning mirror was incorporated to reduce the mechanical footprint, and a long-pass filter (cutoff 650 nm) and a 785 nm notch filter (FELH0650 and NF785-33, Thorlabs, Newton, NJ) were also added in the fluorescence channel to block the excitation laser lines. A visible imaging channel was used to guide imaging and surgery. An achromatic lens (f=60  mm, AC254-060-A, Thorlabs, Newton, NJ) and a 2D RGB color camera (daA1920-160uc, Basler, Ahrensburg, Germany) were used to collect the visible light for white-light reflectance imaging and guidance.

**Fig. 3:**
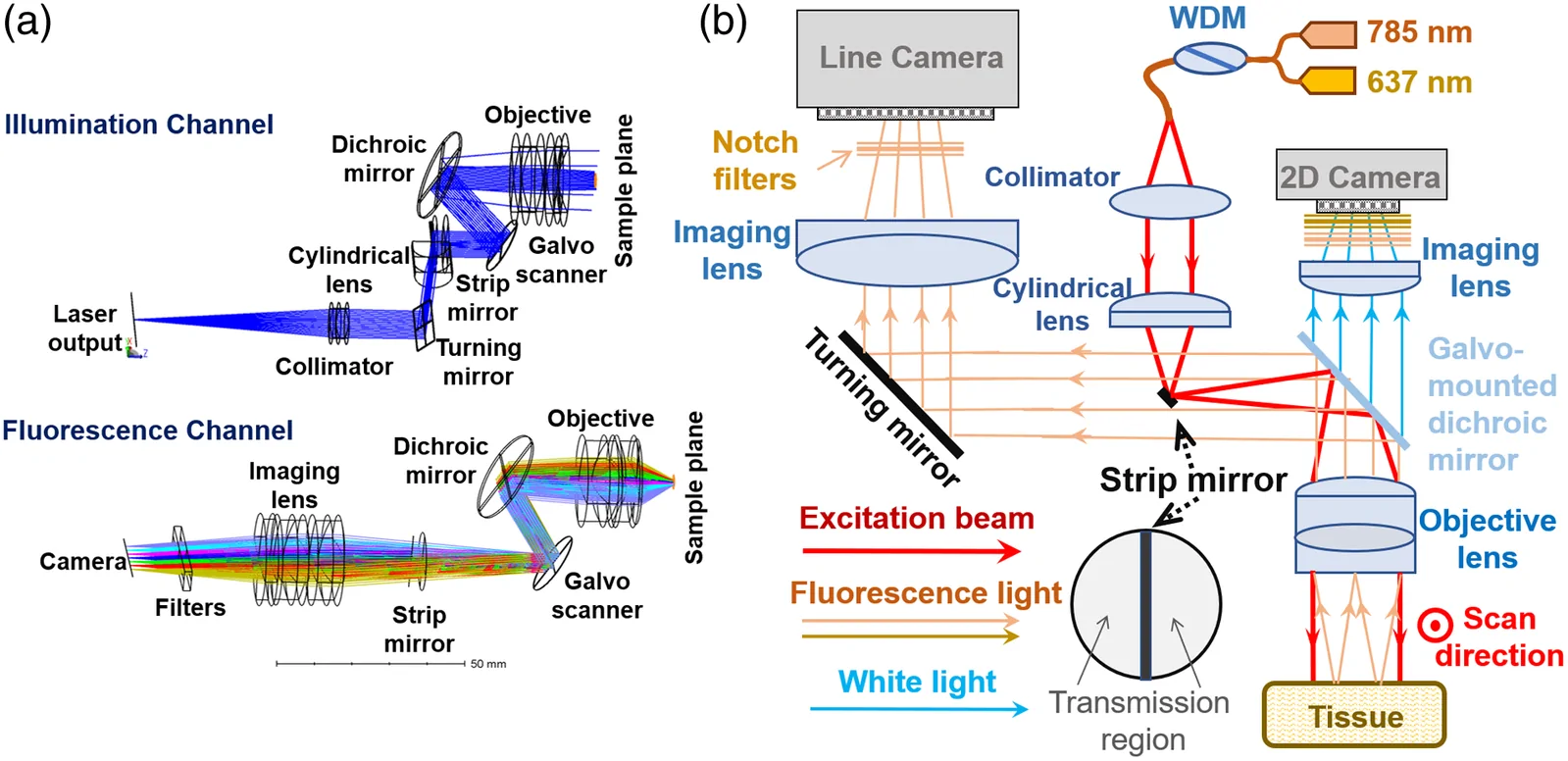
(a) Optical design and (b) schematic diagram of STM fluorescence imager.

A 3D SolidWorks opto-mechanical design and photographs of the STM fluorescence imager housed within a 3D-printed enclosure are shown in Figs. 4(a)–4(c). The dimensions of the STM fluorescence imager are 95×100×135  mm. The imager consists of only off-the-shelf optical components to ensure cost-effectiveness. The mechanical parts of the imager consist of custom-fabricated aluminum and 3D-printed components. A 3D-printed enclosure covers the optomechanical components and supports the forced air heat dissipation elements. In addition, the STM fluorescence imaging head is mounted on an articulating arm, and all other major components, including the excitation lasers, data acquisition/processing unit, and computer, were integrated into a single custom-designed instrument enclosure mounted on a medical-grade mobile cart to enhance maneuverability and positioning during preclinical experiments and future clinical use. This integrated instrument design significantly enhanced system portability and usability [Fig. 4(d)]. The front panel of the instrument enclosure has several control interfaces, allowing for streamlined and reliable cable connections between the imaging head and the control unit.

**Fig. 4:**
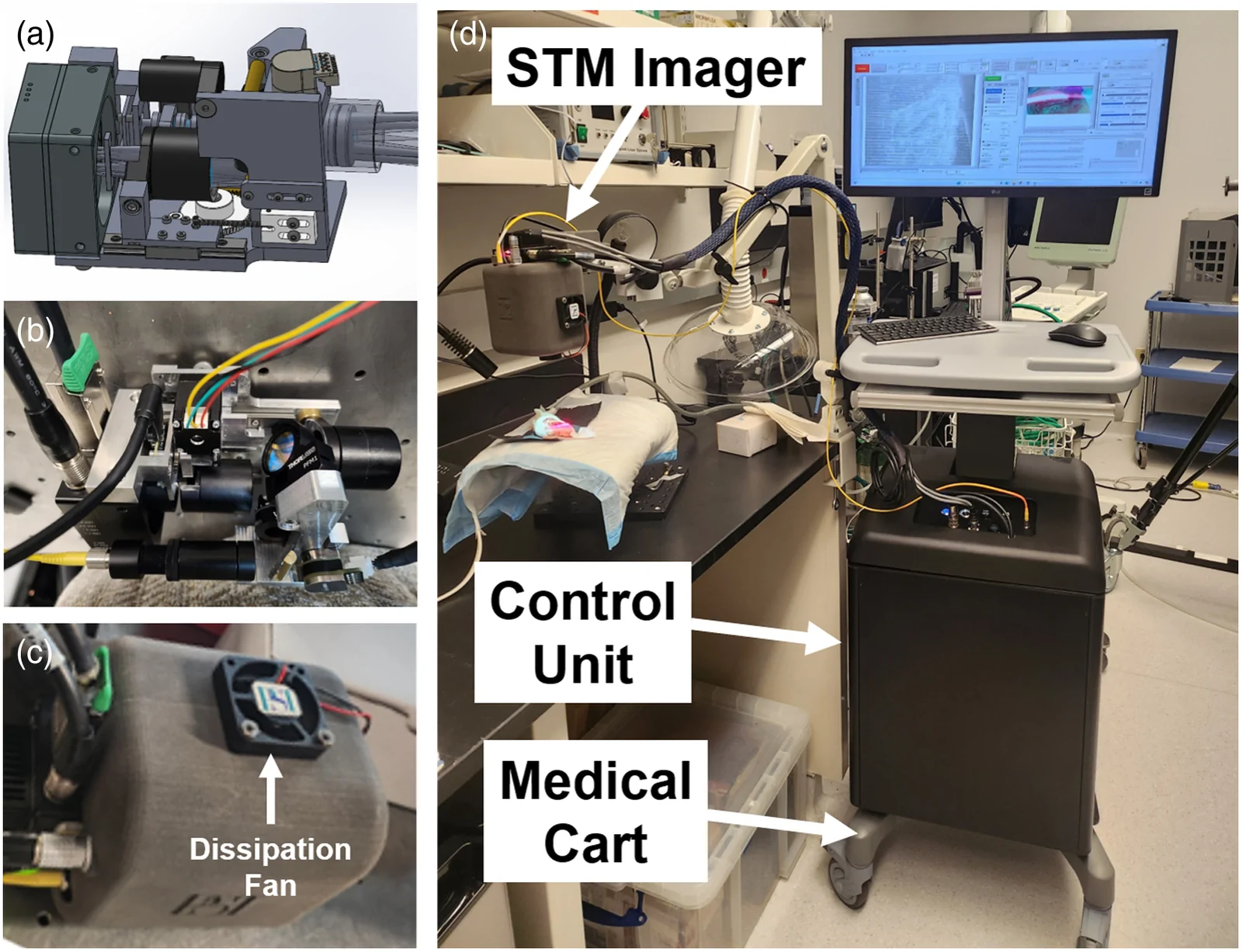
Mechanical design (a) and photographs of the STM fluorescence imager without (b) and with 3D-printed enclosure (c). Custom-designed instrument mounted on a medical-grade mobile cart prepared for *in vivo* animal imaging (d).

### 2.3 STM Instrument Operation Software

A custom graphic user interface (GUI) software program was developed (LabVIEW, National Instruments, Austin, TX) to streamline image acquisition during *in vivo* animal studies and *ex vivo* human specimens imaging (Fig. 5). The major software features include user controls of hardware conditions (e.g., lasers and cameras parameters) to vary critical imaging parameters (e.g., nonmodulation and modulation, and single or dual laser excitation) and defined image acquisition/processing/display modes (e.g., acquisition and display of both visible and fluorescence images). A data acquisition card (PCIe6323, National Instruments, Austin, Texas, United States) is used to control the hardware components. Fluorescence images were acquired using a high-speed frame grabber coupled to a scientific CMOS camera, both provided by Ximea. An automated image acquisition and data-saving function was implemented, enabling sequential image capture under varying instrument parameters, including laser wavelength and STM settings.

**Fig. 5:**
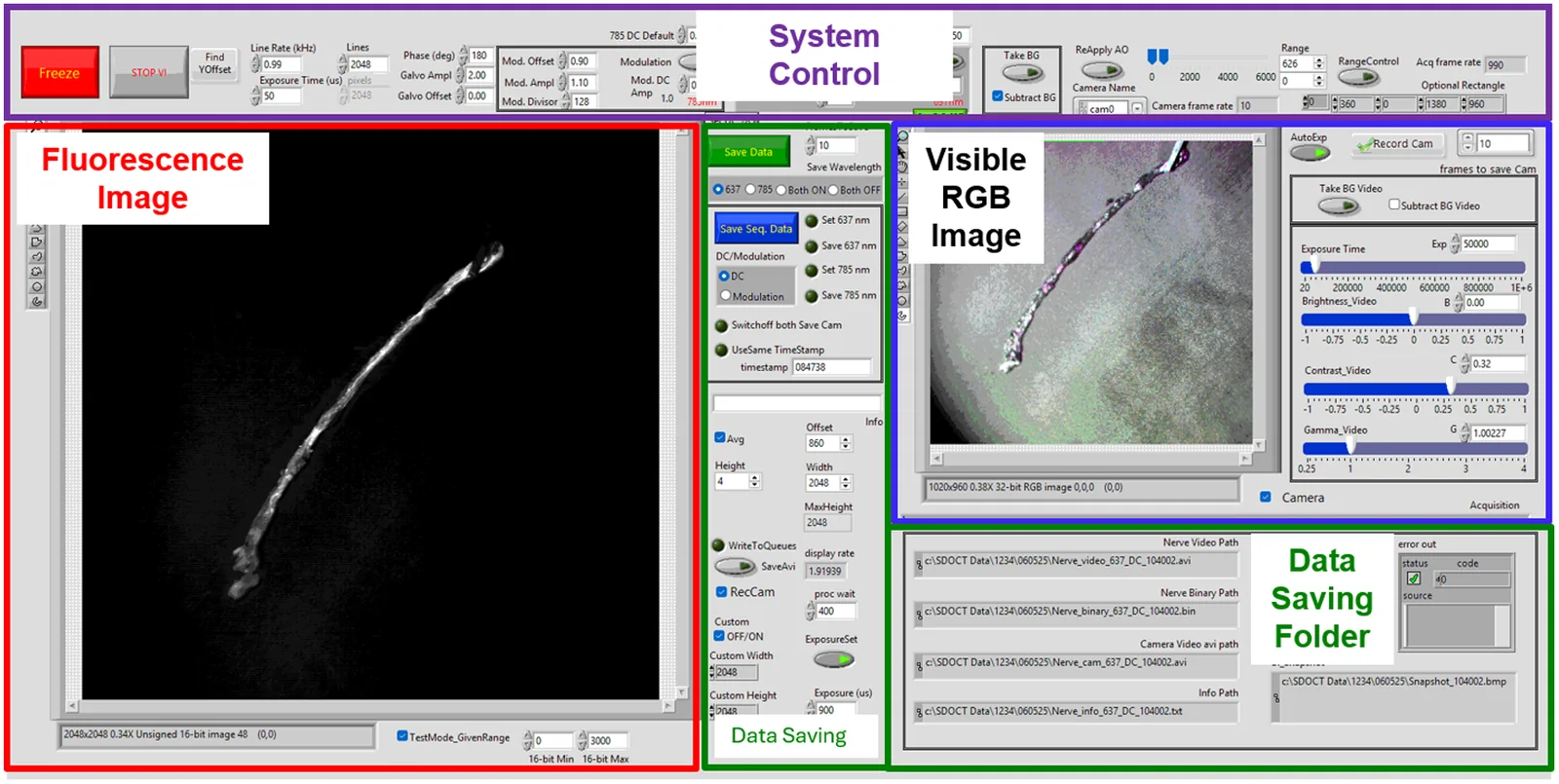
LabVIEW-based user interface of control and data acquisition/processing/display software. 2D displays of fluorescence (red-colored rectangle) and visible (blue-colored rectangle) human nerve images, and control (purple-colored rectangle) and saving panels (green-colored rectangle).

### 2.4 STM Demodulation Algorithm

A 1D amplitude-demodulation-based algorithm was developed to remove the background. The algorithm was integrated into the GUI software using standard LabVIEW functions to enable real-time image processing and display. The algorithm employs time-domain processing (equivalent to 1D Fourier domain processing), with a Butterworth low-pass filter incorporated to reduce image noise. The simplified processing workflow is illustrated in Fig. 6. The algorithm demodulated 2D images line by line, following these steps: (1) Process each image line in the modulation direction, (2) Compute the DC value of each pixel along the line using a low-pass filter, (3) Subtract the DC component from the modulated line profile, (4) Apply a 1D low-pass filter to remove residual modulation frequency, (5) Repeat the process for all image lines, and (6) Apply a 2D low-pass filter to the entire image to further suppress noise.

**Fig. 6:**
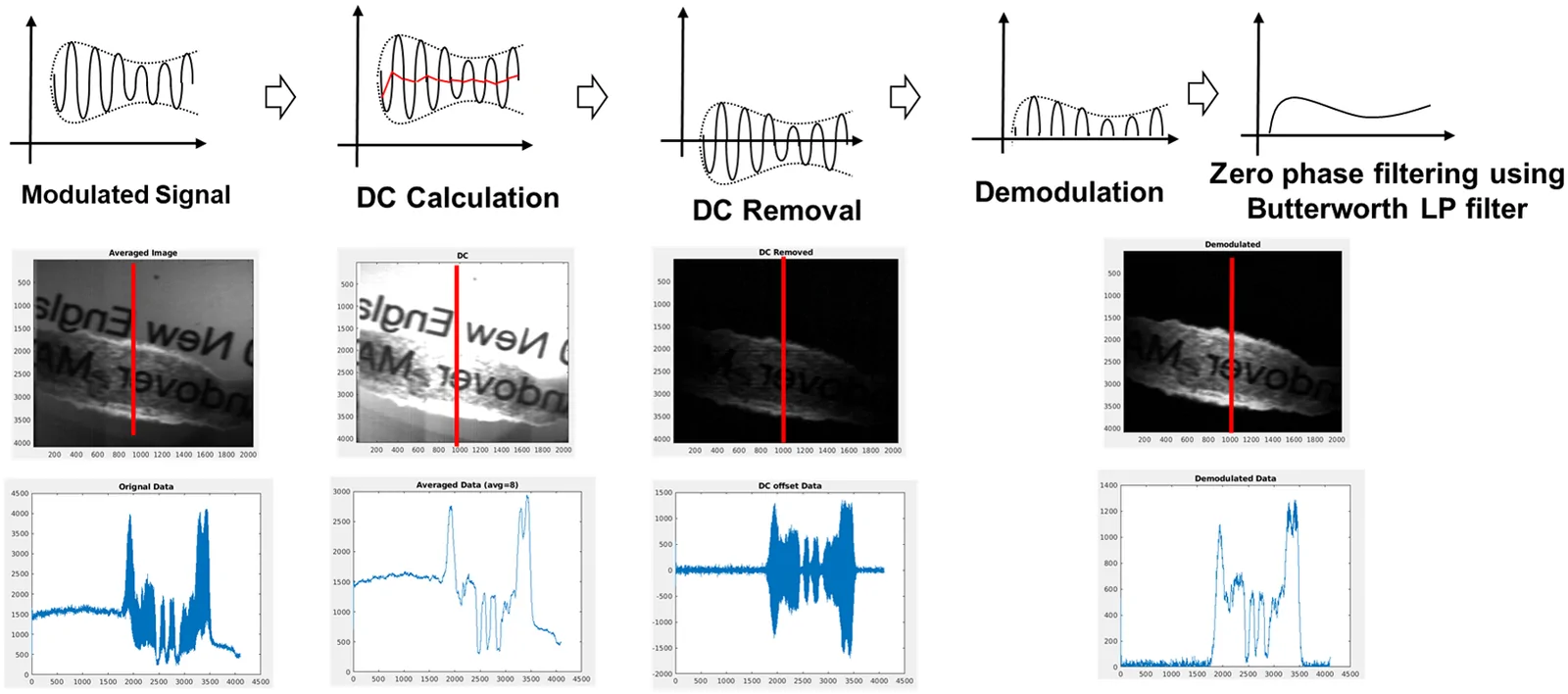
Time-domain demodulation and reconstruction algorithm. Graphs at the bottom represent cross-sectional profiles along the red lines in each corresponding image.

### 2.5 Preparation of *In Vivo* Animal Models and *Ex Vivo* Human Specimens

*In vivo* animal imaging was performed on 30 mice to evaluate the capabilities of the STM fluorescence imager. All procedures were conducted in accordance with the Dartmouth Institutional Animal Care and Use Committee guidelines (Protocol 2279). Equal numbers of male and female CD-1 Institute of Cancer Research stock mice (Charles River Laboratories, Wilmington, Massachusetts, United States) were used. Prior to imaging, mice were fed a purified, chlorophyll-free diet (MP Biomedicals, Irvine, California) for at least four consecutive days to reduce chlorophyll associated autofluorescence in standard feed. On the day of imaging, mice received a weight-based intravenous injection (1.0 to 5.0  mg/kg) of the nerve-specific fluorescent probe LGW16-03 via the tail vein, one hour before undergoing nonsurvival surgery. The surgery involved exposing the sciatic nerve in the right and/or left flank. Following nerve dye injection, the 1-h incubation period, anesthesia induction, and exposure of the sciatic nerve, a weight-based intravenous injection (1.0 to 1.5  mg/kg) of ICG was administered immediately prior to imaging. Dual-wavelength fluorescence imaging was then performed using 637 nm excitation for LGW16-03 and 785 nm excitation for ICG to visualize the exposed sciatic nerve and vasculature in adjacent muscle tissue, respectively. General anesthesia was maintained throughout each non-survival procedure using 1% to 3% isoflurane delivered with oxygen at 1 to 2  L/min [Figs. 7(a)–7(d)]. At the end of the imaging session, mice were euthanized, and sciatic nerve, muscle, and/or adipose tissues were harvested for further *ex vivo* imaging.

**Fig. 7:**
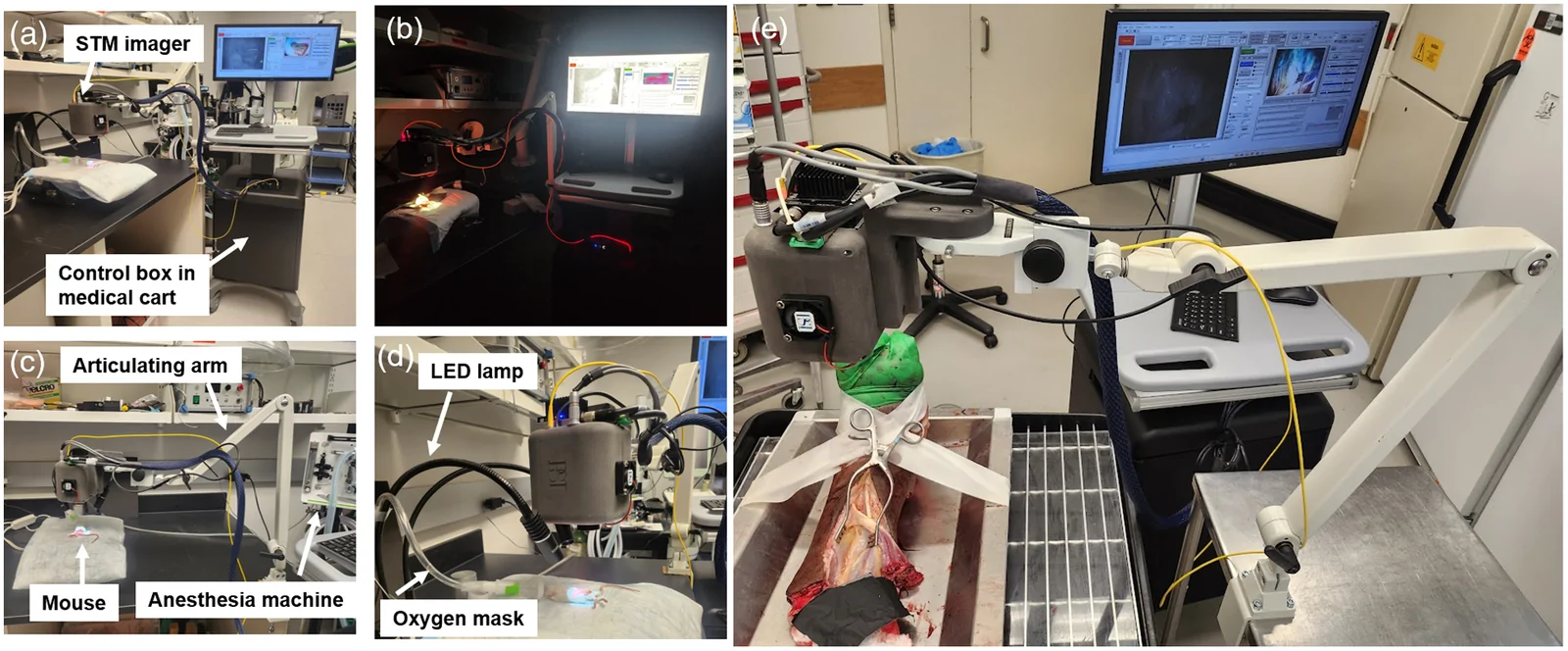
*In vivo* mouse and *ex vivo* human amputated limb experimental setup using the STM fluorescence imager. Photographs of (a) setup with room light on, (b) setup with room light off, (c) STM imager with articulating arm, (d) mouse positioned under the STM imager, and (e) human nerve tissue from an amputated limb.

*Ex vivo* human nerve specimens were obtained according to one of two Institutional Review Board (IRB)-approved protocols at Dartmouth-Hitchcock Medical Center (DHMC) in Lebanon, New Hampshire. Individual human nerve specimens were procured from head and neck cancer resection procedures by a board-certified surgeon (IRB STUDY02000504). Each nerve was isolated by the surgeon from the bulk tissue resection in the OR, placed in saline in a sealed container, and transported to the research laboratory. A small segment of surrounding tissue (e.g., muscle or adipose) in contact with the nerve was also removed to provide a tissue background for comparison. Nerve harvesting in this study did not impact the clinical procedure, resection margins, or any aspect of standard of care. Each nerve was submerged in a fluorophore bath consisting of 0.05  mg/mL LGW16-03 suspended in PBS. Each sample was bathed in fluorophore for five minutes, followed by washing with PBS prior to imaging, in alignment with a previously published workflow.^49^^,^^50^

Human nerves from an amputated limb were also imaged according to an established amputated limb protocol (IRB STUDY00031759). The protocol involved enrollment of patients receiving transtibial or transfemoral amputation due to trauma, infection, or vascular insufficiency; oncological indications were also acceptable if the specimen did not need to be analyzed for margin status. After standard-of-care amputation in the OR, each specimen was transferred to the research laboratory for fluorophore administration and imaging. A previously published, perfused, amputated limb model was used for fluorophore administration.^52^ A dominant artery in the limb (e.g., superficial femoral, anterior tibial, or posterior tibial artery) was identified, transected, and connected to a cardiopulmonary bypass machine (S3, Sorin Stockert, Breisgau, Germany) with a vascular cannula. Each limb was perfused with sterile saline at 90 to 180  mL/min to maintain pressure within a physiological range (<120  mmHg). A needle probe (Stryker, Kalamazoo, Michigan, United States) was used to verify in-line pressure during perfusion. Within 10 min of initial saline perfusion, a standard dose of LGW16-03 was administered intra-arterially (1  mg/mL LGW16-03 suspended in PBS; 10 mL bolus). Fluorophore perfusion continued for 10 min, after which the perfusate was swapped for sterile saline for a 20-min washout. After perfusion, target nerves (i.e., common peroneal and/or tibial) and surrounding background tissues (e.g., muscle, adipose) were exposed for imaging via a cut-down procedure by a board-certified surgeon [Fig. 7(e)].

## 3 Results

### 3.1 Characterization of STM Imager

The SBR enhancement and quasi-confocal effect of the line-scanning STM imaging system were validated using the LGW16-03 dye. First, we measured the SBR of the LGW16-03 dye-stained business card images under both modulation and demodulation. As shown by Fig. 8(a), the STM technique enabled us to achieve high-efficiency suppression of the ambient background without compromising the spatial resolution. Compared with nonmodulated images, the signal-to-background is improved by a factor of ∼100, without resolution degradation.

**Fig. 8:**
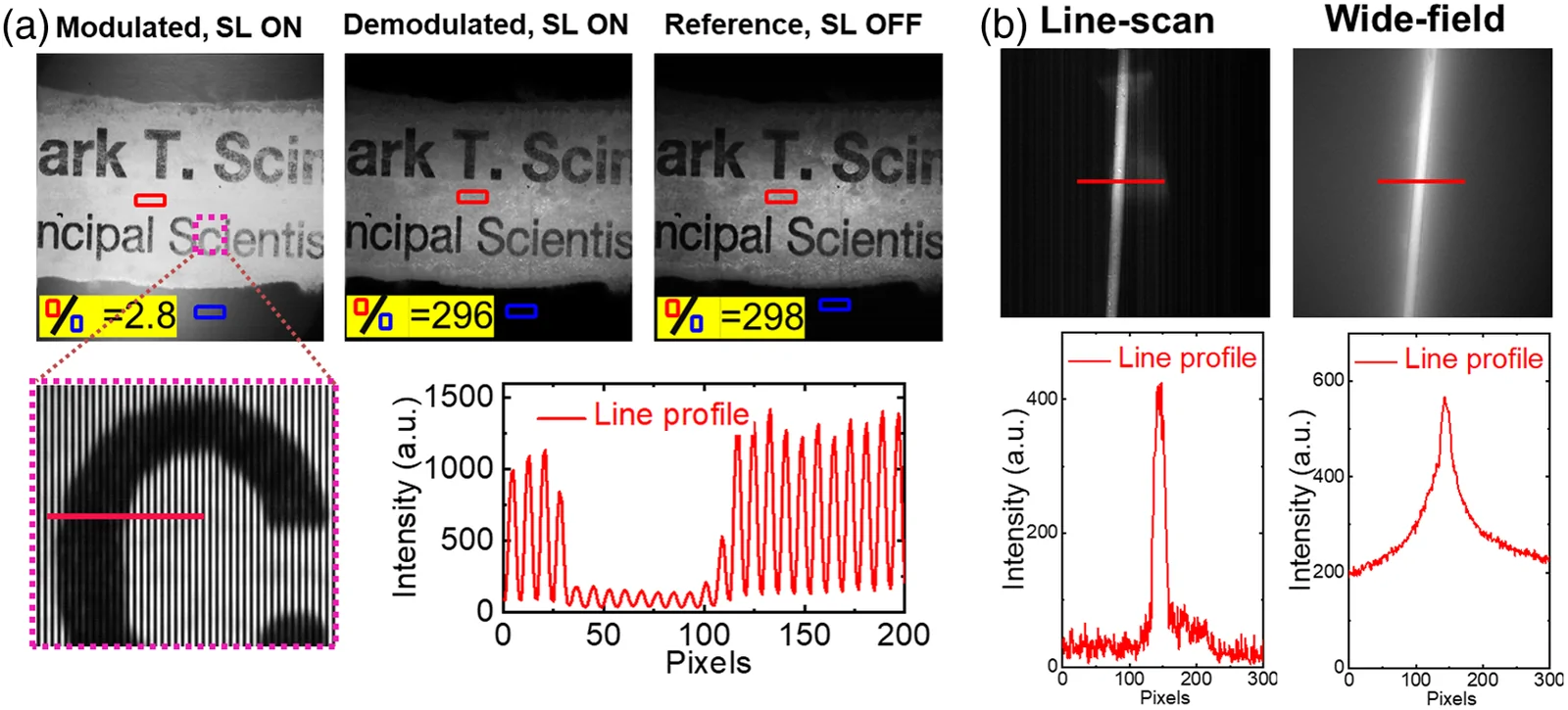
Advantages of line-scan STM fluorescence imager. (a) Spatially modulated and demodulated images of a business card with an LGW16-03 dye-stained horizontal band in the middle. The zoomed-in image and line profiles in the lower row show the high-contrast spatial modulation. SL: surgical lamp of high brightness ($∼8\;\mathrm{mW}/{\mathrm{cm}}^{2}$). (b) Line-scan and wide-field images of a 0.4-mm diameter capillary tube filled with LGW16-03 dye and submerged in a 1% Intralipid solution at 0.5 mm below the surface. The plots below are line profiles of the red lines in the above images, respectively.

To demonstrate the quasi-confocal effect, we used the system to image a capillary tube that was filled with LGW16-03 dye and submerged in a 1% Intralipid solution. As shown in Fig. 8(b), the line-scan imager provides significantly higher contrast and resolution.

Simultaneous two-wavelength fluorescence imaging with the line-scan STM imaging system was demonstrated using simple imaging targets containing both LGW16-03 and ICG. As illustrated in Fig. 9(a), the letters “P” and “I” were painted on a plain paper card using an LGW16-03 solution, while the letter “S” was painted with an ICG solution. A second sample was prepared on a microscope slide with two wells: the left well contained ICG, and the right well contained LGW16-03 [Fig. 9(b)]. Initial images (top row) were captured using simultaneous intensity modulation of the two lasers, with their phases set 180 degrees apart. The demodulated images were then reconstructed (Fig. 9, second and third rows), as it separated the LGW16-03 and ICG signals into their respective channels. A weak signal observed in the ICG well in the 637 nm channel of Fig. 9(b) (second row) is attributed to ICG fluorescence under 637 nm excitation, indicating the high sensitivity and large dynamic range of the imager. False-color merged images are presented in the bottom row of Fig. 9. The imaging results confirm effective spectral separation of the two fluorophores by the STM approach without compromising spatial resolution.

**Fig. 9:**
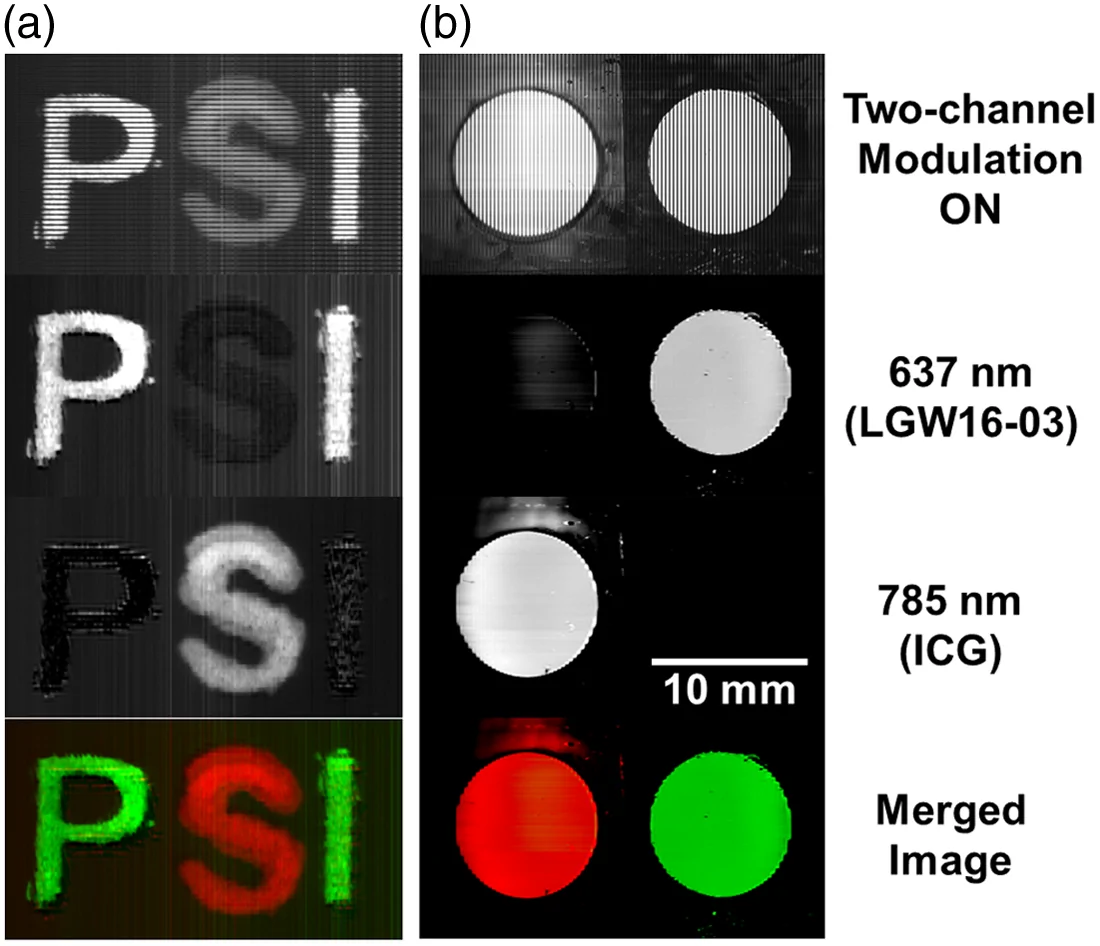
Testing of the STM fluorescence imager with two fluorescent dyes: LGW16-03 for 637 nm and ICG for 785 nm excitations. (a) Letters on paper (LGW16-03: P and I, ICG: S). (b) Two solutions on a slide glass (LGW16-03: right, ICG: left). Scale bar=10  mm.

### 3.2 *In Vivo* Animal Nerve Imaging Capability of STM Fluorescence Imagers

Fluorescence images were acquired during *in vivo* mouse experiments under various conditions, including different lighting (room lights on/off), modulation settings (on/off), and excitation wavelengths (637 and 785 nm). Figure 10 presents representative *in vivo* images of murine sciatic nerve tissue and muscle vasculature captured using the line-scan STM fluorescence imager. In the raw modulated images, strong background signals from the LED lamp ($∼11\;\mathrm{mW}/{\mathrm{cm}}^{2}$) were effectively removed via time-domain demodulation, resulting in high-contrast reconstructed images. The demodulated images clearly highlight nerve tissue and blood vessels [Figs. 10(a) and 10(c)]. Fine nerve structures (green arrow) excited by 637 nm laser fluorescence were visible in the demodulated image [Fig. 10(a)] as confirmed in the corresponding visible RGB image [Fig. 10(b)]. However, blood vessels (red arrow), not visible in the visible RGB image, were clearly delineated in fluorescence images acquired with 785 nm excitation [Fig. 10(c)]. The two fluorescence channels were fused into a pseudo-colored composite [Fig. 10(d)], demonstrating accurate co-registration of the two imaging channels. These results confirm that the STM imager provides high-contrast visualization of nerve and vascular structures, even under intense background illumination from high-brightness LED lighting ($∼11\;\mathrm{mW}/{\mathrm{cm}}^{2}$), which is brighter than typical surgical lamps ($<5\;\mathrm{mW}/{\mathrm{cm}}^{2}$).

**Fig. 10:**
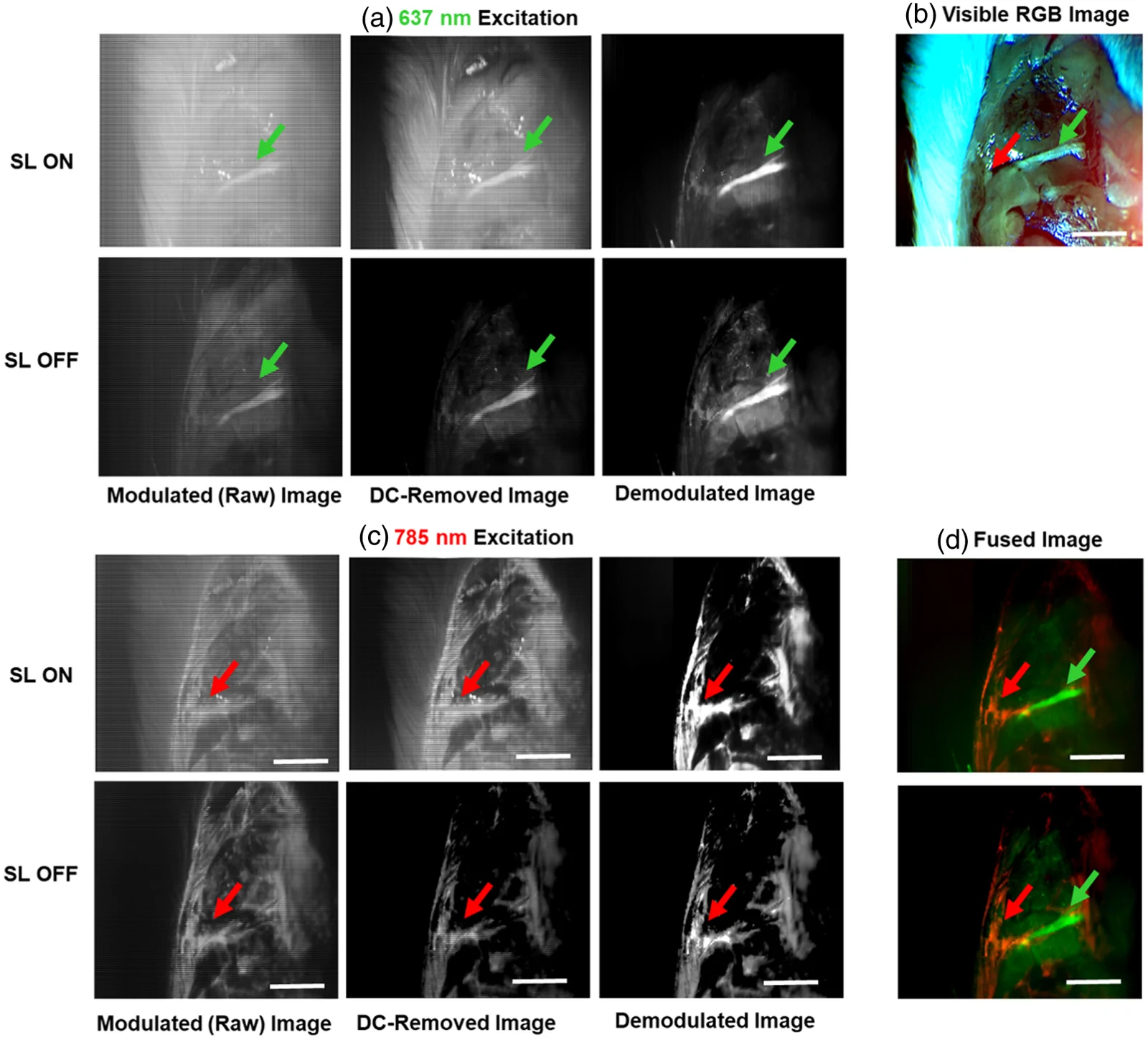
*In vivo* images of a murine sciatic nerve and blood vessels. Modulated (raw), DC-removed, and demodulated two-wavelength fluorescence images captured by the line-scanning STM system (a, c) and a wide-field color camera image for comparison (b). The fluorescence images were obtained by excitations of 637 and 785 nm lasers while the surgical lamp (SL) is on or off. Two-channel images were merged in (d). Scale bar=0.5  cm.

### 3.3 *Ex Vivo* Human Nerve from Otolaryngology Specimens

Line-scan STM fluorescence images were acquired of *ex vivo* human nerve specimens under various acquisition conditions, including different lighting configurations (room light on/off) and modulation settings (on/off). Figure 11 shows images of the transverse cervical nerve, a sensory nerve, captured using the STM imager. A surgical clip identifies the proximal end, while a circular mass of surrounding tissue (muscle and fat) remains attached at the distal end. The specimen, labeled with LGW16-03, was excited with the 637 nm laser. ICG was not administered, as no blood flow information was needed in these experiments. Imaging was performed under strong ambient light ($∼10\;\mathrm{mW}/{\mathrm{cm}}^{2}$) from an overhead LED lamp, using modulation-based acquisition mode. In the visible RGB image [Fig. 11(d)], the nerve (green arrow) is indistinguishable from the background tissue (red arrow). In contrast, the demodulated fluorescence image [Fig. 11(c)] after background removal showed significantly improved contrast relative to the surrounding muscle and fat tissue. Imaging was also performed under the room-light-off condition [Figs. 11(e) and 11(f)]. The final demodulated images clearly resolve the nerve independent of room lighting conditions.

**Fig. 11:**
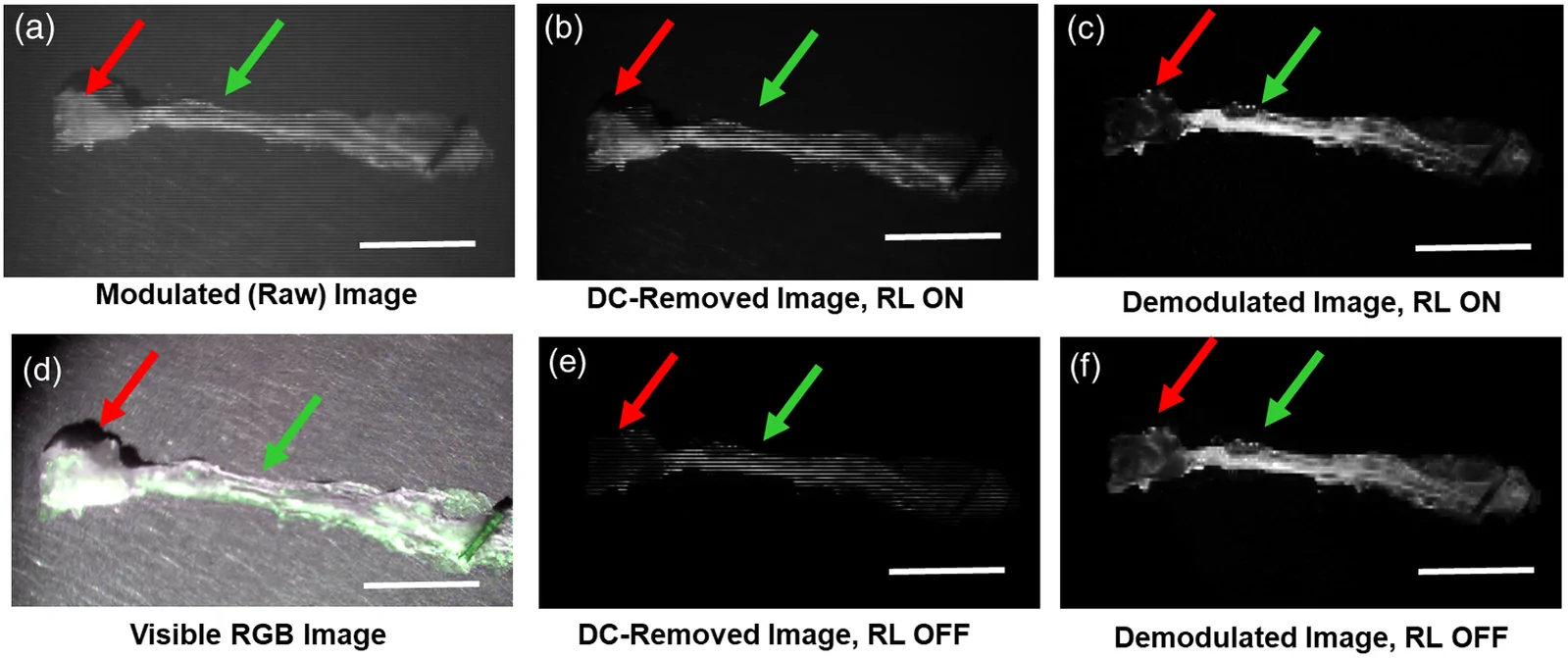
*Ex vivo* imaging of human transverse cervical nerve specimen using the STM fluorescence imager under strong room light conditions. Scale bar=0.5  cm. RL: room light.

### 3.4 *Ex Vivo* Human Nerve Imaging in an Amputated Limb

STM fluorescence imaging of intact human nerve tissue in an amputated limb was performed using two different modes by switching the objective lenses, i.e., large field of view mode designed for human imaging and small field of view mode designed for small animal imaging (Fig. 12). The key parameters distinguishing the two modes were: field of view (FOV) ($125\;{\mathrm{mm}}^{2}$ for human versus $25\;{\mathrm{mm}}^{2}$ for animal), spatial resolution (SR) (52  μm for human versus 15  μm for animal), and working distance (WD) (240 mm for human versus 75 mm for animal). Figure 12 shows representative visible RGB and demodulated fluorescence images from both modes with the room light on. Although a board-certified surgeon was able to locate the nerve within the surrounding muscle and fat tissues, general nurses and researchers were unable to identify the nerve using either physical observation or the RGB images. In contrast, the demodulated fluorescence images clearly revealed the nerve (green arrow) with high contrast [Fig. 12(a)]. The nerve was even more distinctly visualized in the small FOV mode, which provided higher spatial resolution [Fig. 12(b)]. These findings highlight the significant advantages of the STM fluorescence imaging technology in visualizing hidden nerve structures, even under strong ambient lighting. This capability is particularly valuable for FGS, helping avoid accidental nerve injury. Several additional high-intensity regions were observed in the demodulated image. These regions were not anatomically confirmed as nerve tissue and may represent nonspecific fluorophore retention in surroundings. Because STM suppresses ambient illumination but does not distinguish nerve-specific fluorescence from modulated nonspecific tissue fluorescence, further anatomical or histological validation is required to identify these signals.

**Fig. 12:**
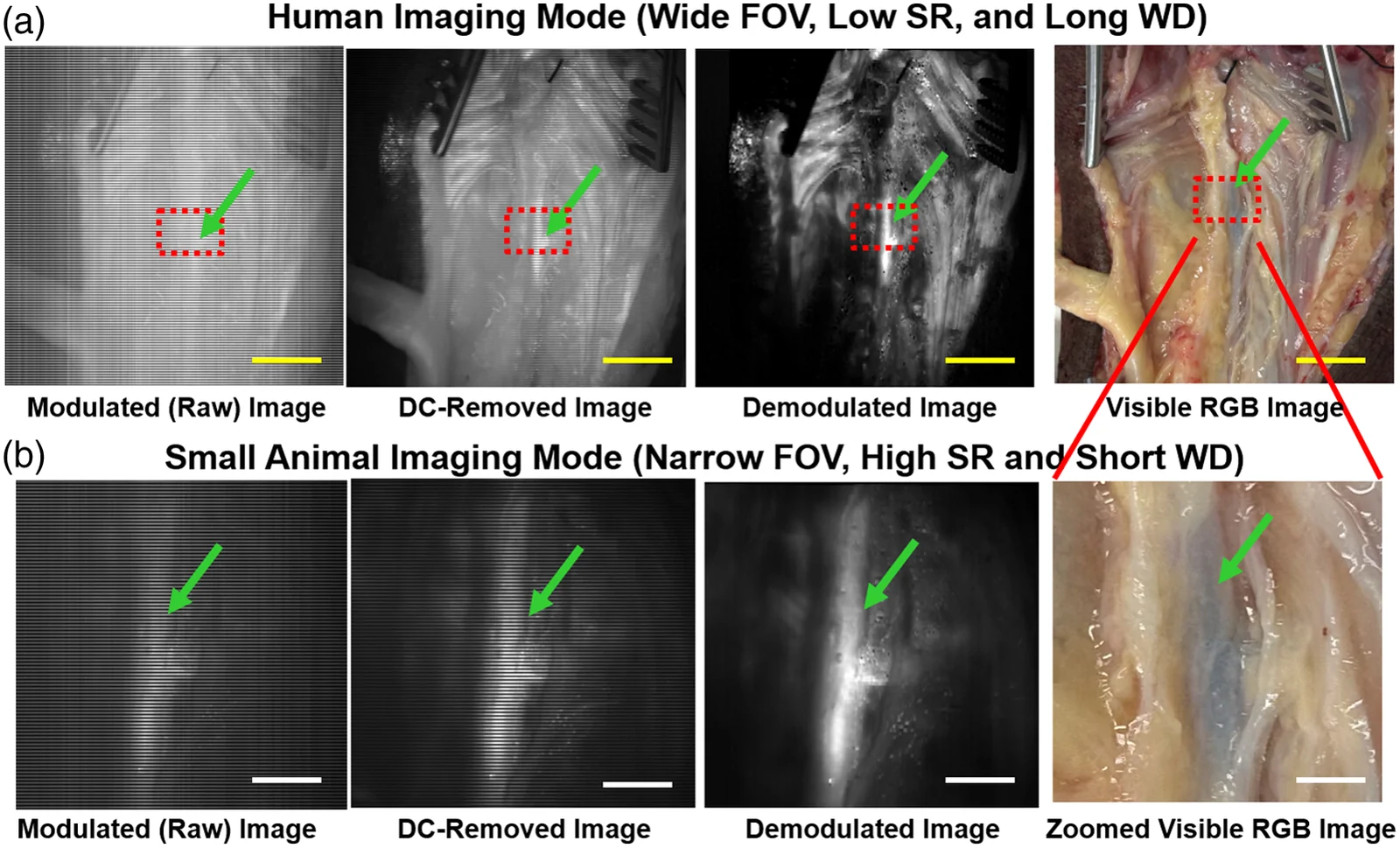
*Ex vivo* imaging of a human nerve in an amputated limb using the STM fluorescence imager. (a) Large FOV imaging mode, and (b) small FOV imaging mode. Yellow scale bar=2.5  cm. White scale bar=0.5  cm.

## 4 Discussion and Conclusion

### 4.1 Mechanisms Underlying Background Suppression and Contrast Enhancement in Line-Scanning STM Imaging

Line-scanning STM technology offers multiple advantages that overcome the limitations associated with standard 2D imaging. First, it enables high-frequency and high-contrast modulation by varying the amplitude of the excitation laser in time. As illustrated by Fig. 1(b), a sinusoidal amplitude modulation is converted into spatial modulation in the final 2D image. In this case, the modulation depth of the fringes is unaffected by the optical transfer function of the imaging system or by tissue optical properties. Moreover, enhanced image resolution can be achieved by oversampling or increasing the number of lines used to reconstruct each image. When combined with the 2048-fold rejection of ambient light provided by the line-scan approach [including the ∼100 × rejection shown in Fig. 8(a)], the STM imagers reduce ambient light background interference by more than five orders of magnitude under optimal conditions. The limit of background suppression for spatial modulation is determined by the dynamic range of the camera. Line-scan cameras with a dynamic range of 12 or 14 bits are commercially available and theoretically offer background rejection factors of 2048 or 4096, respectively.

Second, the line-scan imaging approach forms a quasi-confocal setup.^51^ This confocal effect rejects multiply-scattered photons, which would otherwise reduce the image contrast or SBR. We note that the drawback of the confocal imaging setup is the limited penetration depth in tissue, due to the low level of ballistic light signal retained in the reconstructed image.

In addition, the line-scan approach essentially forms a time-gated imaging scheme. Due to the sequential scanning, the line-scan camera is exposed at each spatial location for a short time period (line-scan camera exposure or dwelling time of the line focus). In contrast, the 2-D camera continuously samples every location (i.e., pixel) during the camera exposure time. The exposure time of the line-scan camera was set to <50  μs, which is approximately two orders of magnitude shorter than that of the 2-D camera (e.g., 3 ms). Moreover, this short exposure time did not reduce the signal level, because the line illumination remains at the line focus for the same duration. Therefore, although the excitation light is constant in time, the duty cycle of the line-scan-based 2-D imaging is 1/N, where N is the number of lines for each image. For 2048 lines per image, this leads to a background rejection rate of 1/2048.

### 4.2 Current Limitations and Mitigation Strategies

The current STM approach requires further refinement to address several practical and translation limitations for clinical implementation.

(a) Penetration depth: The quasi-confocal, visible-wavelength design prioritizes contrast over imaging depth, enabling reliable nerve visualization of exposed or near-surface nerves. However, the confocal mode has limited imaging depth, and its performance diminishes in highly scattering adipose or fibrotic tissue.
(b) Motion artifacts: As a scanning-based imaging approach, the STM imager is sensitive to sample motion. In the current configuration, four camera rows are used at each line-scan position to maximize signal collection, resulting in an imaging rate of ∼18 frames per second (fps). By reducing the acquisition to a single camera row (or one line), the system can achieve 2D imaging rates of up to 70 fps, thereby reducing motion-induced artifacts. Additional improvements, including optimization of the high-speed acquisition mode and implementation of motion-correction algorithms, are under development.
(c) Latency: Demodulation, reconstruction, and display of each fluorescence image are completed within ∼100  ms after completion of the raster scan. At the current image acquisition rate of 18 fps, the total time from the beginning of image acquisition to display of the demodulated image is ∼160  ms, or ∼6  fps, which is sufficient for intraoperative guidance under stable imaging conditions. Ongoing optimization aims to further increase frame rate and reduce the image processing and display latency.
(d) Fluorophore dependency: The STM approach is fundamentally modulation- and detection-based and is not bounded to specific fluorescence wavelength. Therefore, it is broadly compatible with other nerve-labeling agents consistent with the excitation/emission wavelengths of the system that can be custom designed. While the current study demonstrates performance using LGW16-03, the system can be readily adapted (e.g., excitation source and emission filtering) to support other fluorophores such as DSB and oxazine derivatives, and future work will include validations using multiple agents.
(e) Validation of human data: *Ex vivo* and perfused amputated limb models may not fully replicate *in vivo* surgical conditions, including patient motion, active blood flow dynamics, and tissue variations. However, the perfused limb model provides a clinically relevant intermediate step that preserves vascular perfusion and tissue optical properties, enabling evaluation under realistic OR lighting. Ongoing and future studies are focused on *in vivo* validation to further assess system performance under dynamic surgical conditions.

### 4.3 Summary and Future Direction

We have demonstrated a novel intraoperative fluorescence imager that can acquire high-contrast nerve-specific images via dual-wavelength fluorescence imaging under standard OR lighting. The imager employed an STM approach to reject the background interference from the surgical operating room lighting. The total background rejection rate of the STM fluorescence imager is anticipated to >100,000 under optimal imaging conditions. The improvements were attributed to >2000 from time-gating and >100 from spatial modulation. The capability of the imager was validated through preclinical testing *in vivo* preclinical testing in mice, and *ex vivo* human nerve imaging. The efficient background rejection enabled the STM fluorescence imager to capture high-contrast nerve-specific fluorescence images under standard room lighting.

Future work will focus on advancing technology towards clinical translation, while also expanding research applications. The imager will be experimented across a wider range of animal models with different nerve-targeted contrast agents. Additional future plans will include AI-driven image processing for nerve detection, shared imaging repositories, and quantitative analysis tools.

## Data Availability

Data underlying the results presented in this paper are not publicly available at this time but may be obtained from the authors upon reasonable request.
